# Composing for acoustic robots: Instant synthesis for computer-controlled acoustic instruments through live coding and AI – A research conclusion

**DOI:** 10.12688/openreseurope.20968.1

**Published:** 2025-08-04

**Authors:** Alexandros Drymonitis, Marinos Koutsomichalis

**Affiliations:** 1 Multimedia and Graphics Arts, Cyprus University of Technology, Limassol, 3036, Cyprus

**Keywords:** Instant Synthesis, Instant Composition, Live Coding, Live Scoring, Artificial Intelligence, Robotic Instruments

## Abstract

This report presents the Postdoc research of the first author, titled *Composing for Acoustic Robots: Instant Synthesis for Computer-Controlled Acoustic Instruments Through Live Coding and AI*. This research focuses on instant composition for computer-controlled acoustic instruments through the LiveLily system. Inspired by earlier research by the first author, where music in the style of Bach was created live with the aid of AI, this research took this instant composition approach further, to include original music and combine that with robotic acoustic instruments. The research has been split in two parts, where in the first part a series of short instant compositions was created for a Yamaha Disklavier robotic piano and human performers, while the second part focused on a MIDI-controlled church organ. This split was an intentional method to enable us to test our system in a concert setting and fine tune it according to how it performed, so we could get better results in the second concert. The results of this research are original instant compositions for these two instruments, part of which we present in this paper. Through this process, we developed a system for creating traditional Western-music scores for computer-controlled acoustic instruments live, through live coding and AI, as a way to create instant compositions while maintaining a steady pase for the music content generation.

## Introduction

Composing for Acoustic Robots is the title of the Postdoc research of the first author at the Cyprus University of Technology, with the second author being the scientific supervisor. It commenced in July 2024 and ended in May 2025. It concluded in a series of short instant compositions for a Diskalvier robotic piano and another instant composition for a MIDI-controlled acoustic church organ. The series of short compositions was realised in November 2024. The second composition, for a MIDI-controlled church organ was realised at the beginning of May. This research aims to address the following three questions:

- How can state-of-the-art text-based AI frameworks enhance live coding?
- How can such an AI framework be trained, and how can an effective training dataset be assembled?
- How can an ethical use of AI in the arts be promoted and communicated?

**Figure 1.**
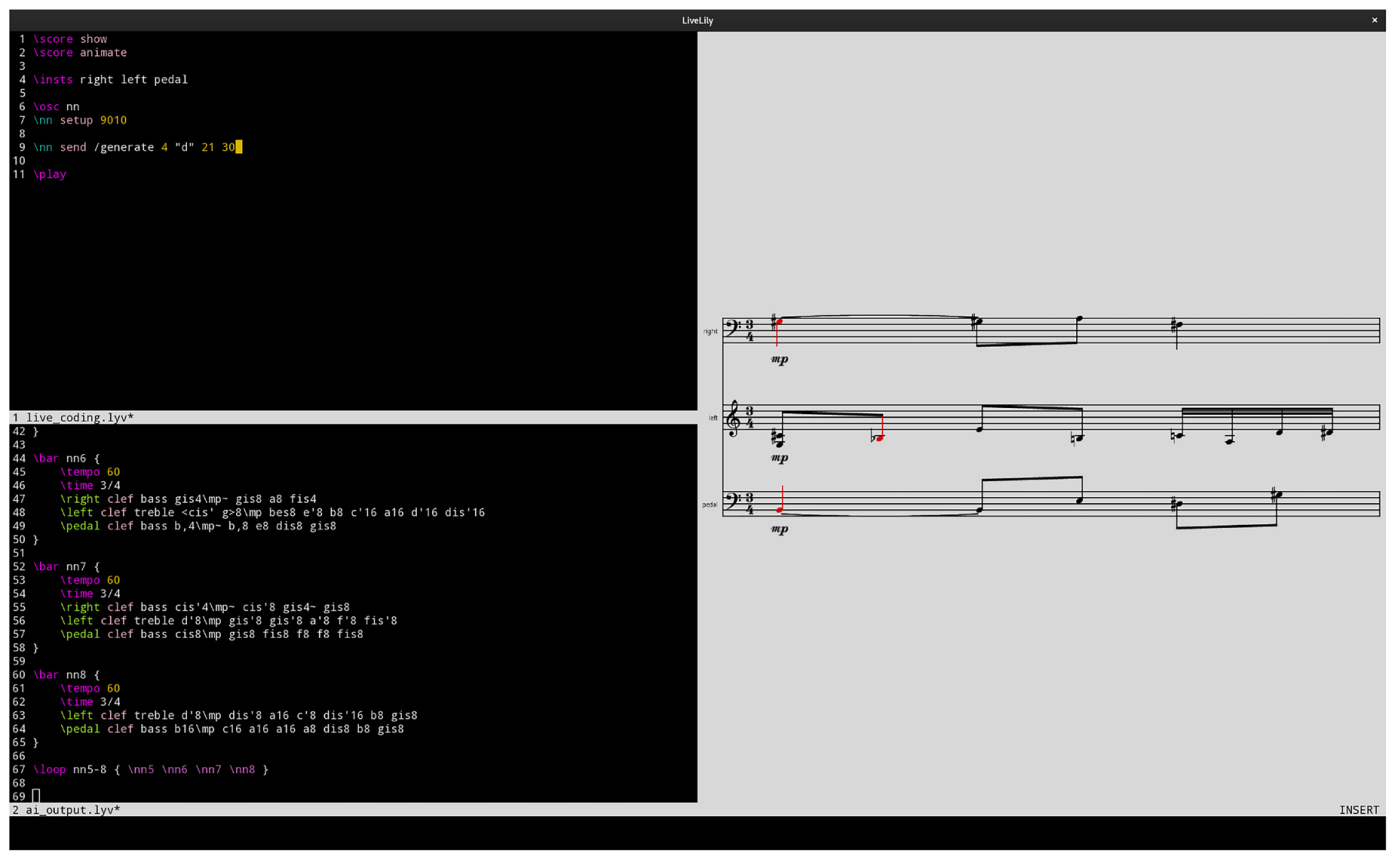
A LiveLily session.

The aim of this research was to produce instant compositions for computer-controlled acoustic instruments, also played by human performers. It brought together three fields of artistic practice, namely live coding, live scoring, and AI in music creativity, equally focusing on each. Live coding and live scoring were employed through the use of the LiveLily system, developed by the first author ( Drymonitis, 2023a; Drymonitis, 2025a). Figure 1 shows a LiveLily session. The *Technology used* section elaborates on the use of AI in this research.

The initial idea for this research was a research by the first author, where the LiveLily system was used with a character-level text generator that was trained on the chorals of Bach, to generate music content in the style of Bach ( Drymonitis, 2023b). This initial research led the first author to initiate the Postdoc research mentioned in this paper, to further investigate and develop this instant composition approach. The idea though transformed to include original music, rather than try to imitate a composer like Bach.

The two iterations of this research – the Disklavier short instant composition series and the instant composition for the MIDI-controlled church organ – comprise the macro-methodology, where each iteration comprises a micro-methodology. These are analyzed in the *Methods* section of this paper. This approach helped determining the various techniques and technology that is needed to realize this research. The main difference between the two iterations is the way the AI was employed. This concerns both the architecture of the AI models that were used, but also the way the training dataset was assembled. While details on the AI technology of the first iteration can be found in an article by the authors of this paper ( Drymonitis & Koutsomichalis, 2025), this report mainly focuses on the second iteration, with only references to the first, for the sake of comparison.

## Technology used

Both iterations of this research are based on the LiveLily live scoring system. LiveLily has been developed by the first author as a system that enables live scoring through live coding in a language inspired by the Lilypond language, developed by Nienhuys and Niewenhuizen ( Nienhuys & Nieuwenhuizen, 2003). This research focuses on instant composition. For this reason, AI has been employed, to enable the creation of music content fast, addressing the issue of slow evolution in live coding performances, as pointed out by Magnusson, where he states that "there is little fun in watching a stressed programmer designing algorithms for minutes before a simple sine oscillator is applied in the playback of a silly melody" ( Magnusson, 2011, p. 503). Besides this constant flow of music content we are trying to achieve, the use of AI provides variations on the original content with which it is being prompted or trained, enabling instant composition performances of longer duration.

In the first iteration of this research, code from the Classical Piano Composer ^1^ project was used, as a way to create harmonic content. This means that the only parameter that was generated by AI was the pitches of the score. The rhythmic part was not assigned to AI at all. While the results of the AI note generation were satisfactory enough to present in a concert, it was rather evident that this was the case mainly because of the compositional style in the pieces that were used to train the AI. The training dataset comprised of four original compositions, created for this project. The style of these composers was mainly atonal, which allowed for a certain amount of randomness in the harmonic content to be acceptable. Additionally, the code was setup in a way that the live coder could provide a percentage within which the AI generated notes would be used, whereas the rest of the notes were drawn from the original compositions.

In the second iteration of this research, the AI used changed completely. The Classical Piano Composer code was not used at all, as this was replaced by the Notochord project by Shepardson *et al.* ( Shepardson *et al.*, 2022; Shepardson & Armitage, 2025) developed at the Intelligent Instruments Lab ^2^, which we collaborated with for the second iteration of this research. This AI model is designed for interactive improvisation using the MIDI protocol. It has been trained on the Lakh MIDI dataset ^3^ which consists of 176,581 unique MIDI files. The advantage of using this model instead of one previously used is twofold. On one hand, this is an already trained model, so we did not have to go through the process of compiling a training dataset and training the model. On the other hand, being designed for interactive improvisation, it is very fast and responsive, making it suitable for creating new musical content in a live performance setting.

Notochord is a Recurrent Neural Network (RNN) written in Python that uses the PyTorch module. To integrate it to the LiveLily system, a Python script was written that receives OSC messages from the LiveLily system, with the number of bars to generate, the name of the composer whose piece is to be used as a feed to the network, the bar number of the piece used as a prompt, and the number of notes to generate for each bar ( Drymonitis, 2025b). This last information in the OSC message sent to the Python script served as a control for the density, and as a result, the difficulty of the generated content. Even though we did not have to assemble a training dataset for the Notochord, we still used original compositions as a way to have variety in the feed prompts we used with the AI. Still, the number of compositions in this second iteration was smaller, and of less importance to the actual AI, since they were not necessary for training the model. The original pieces used in this iteration where composed by the first author and Thanos Polymeneas Liontiris.

While the Notochord model provides content that is coherent from the perspective of pitch, quantization on the time axis was necessary. For each MIDI event, this model provides a MIDI note number, its velocity, and the delta time of its occurrence since the last MIDI event, in seconds. This delta time can be rather arbitrary, even when fed with a prompt of a steady rhythm. For this reason, a sixteenth note grid quantization was applied. Once the model's output was quantized, it was checked for correct total duration for each generated bar of music, and then it was translated to LiveLily strings so they could be fed to the live scoring system. These strings are sent to the LiveLily system via OSC, a feature that enables remote live coding on this system. This way, AI-generated scores are created live during the performance.

## Methods

**Figure 2.**
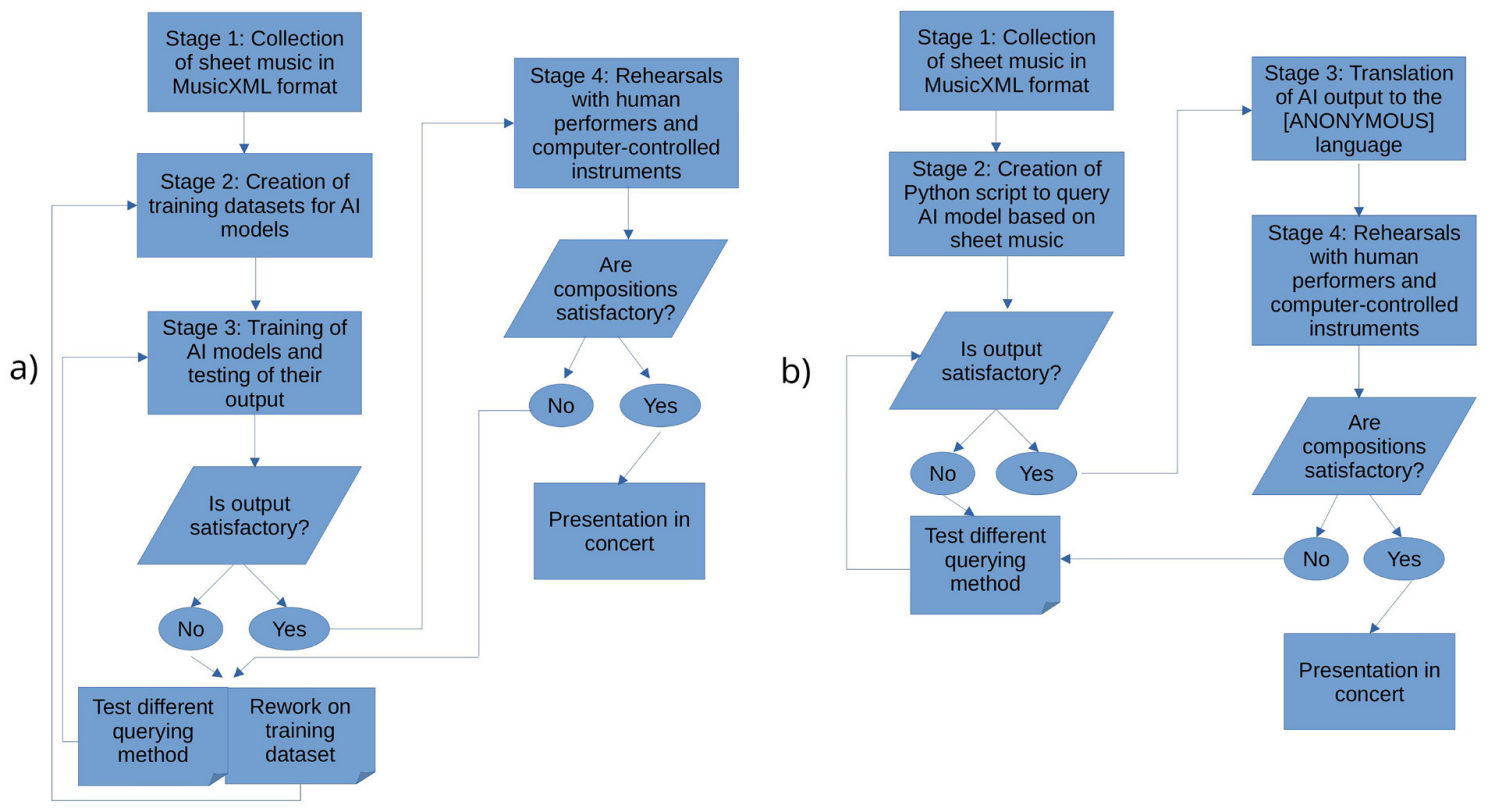
Micro-methodologies. **a)** micro-methodology of first iteration, **b)** micro-methodology of second iteration.

**Figure 3.**
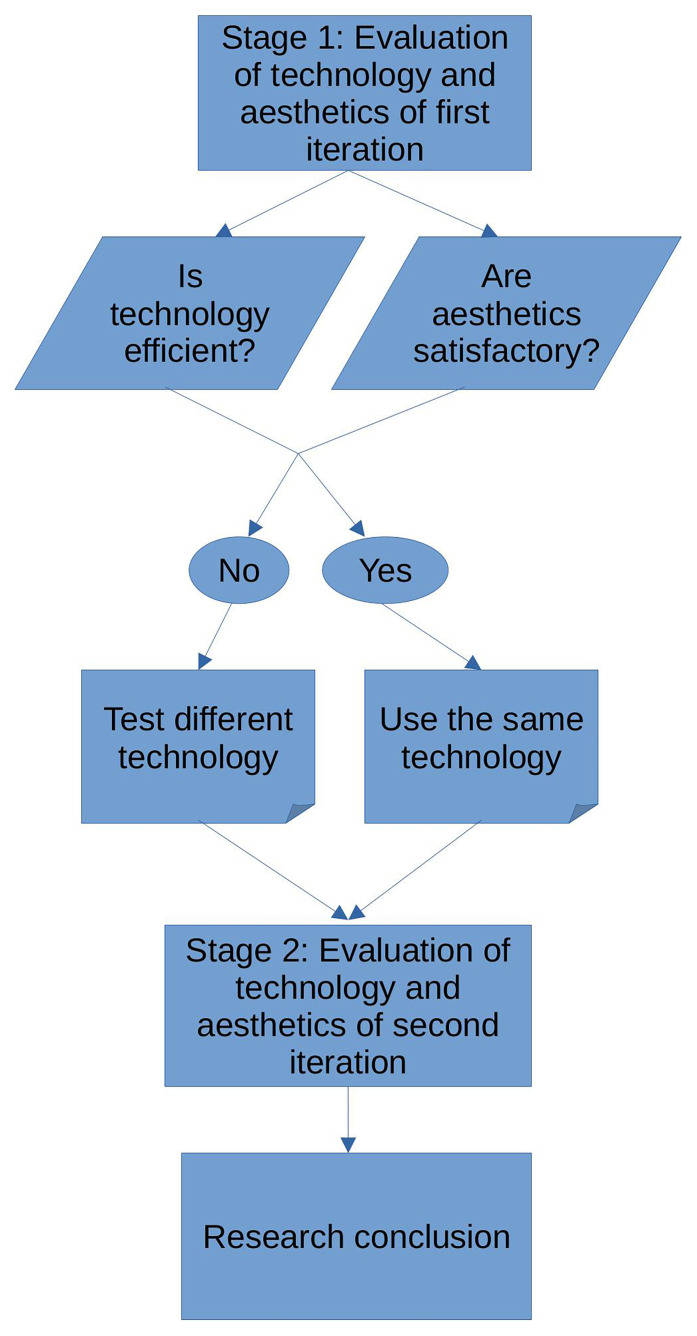
Macro-methodology.

As mentioned in the *Introduction* of this report, the methodology of this research is split into micro-methodology and macro-methodology. This approach was planned from the start, as this postdoc research was designed to realize two different compositions in two concerts. At the beginning of this research, the first micro-methodoloy and the macro-methodology were laid out, while the second micro-methodology was laid out after the first iteration of this research had been realized. Figure 2a illustrates the micro-methodology of the first iteration, Figure 2b illustrates the micro-methodology of the second iteration, while Figure 3 illustrates the macro-methodology.

The main difference between the two micro-methodologies lies in the assembling of a training dataset for the AI and the actual training process, where in the second iteration no such process took place. Instead of collecting pieces and assembling a training dataset, the first stage of the second micro-methodology consisted of the collection of fewer pieces that were used only for querying. The first micro-methodology could possibly roll back to two stages, depending on the performance of the AI model, while the second micro-methodology could roll back to only one stage, since no assembling of a dataset or training took place.

Another difference between the two micro-methodologies was the design approach, where the second micro-methodology was designed with slightly different methods than the first. These methods concerned how the AI performance was tested. In the second stage of the second micro-methodology, a first version of the Python script that communicated with the LiveLily system through OSC and queried the AI model was developed. In this stage, the model's output was sent straight to the MIDI-controlled organ, without the LiveLily system intervening in this process, so as to test its performance. Such an intermediate stage was not used in the first micro-methodology, but was proved necessary by the first iteration of this research.

Once the AI model provided satisfactory results, we entered the next stage, where the Notochord output was translated back to the LiveLily language. This happened in the same Python scripts that did the AI queries. The LiveLily system can be controlled remotely, where it can receive single characters or entire strings via OSC, and these will be typed into its editor. This way, the translated LiveLily strings were sent from the Python script to LiveLily via OSC, and new music content was created.

The last stage of the two micro-methodologies is the same. In this stage, rehearsals with the MIDI-controlled church organ and a performer playing on the same instrument were realized. Even though the AI output had already been tested, this stage helped determine whether the AI-generated music content was within the playing capacity of the performer, in a sight-reading setting. Once the AI-output was tuned to fit the sight-reading capabilities of the performer, we moved on to present this project in a concert.

## Results

**Figure 4.**
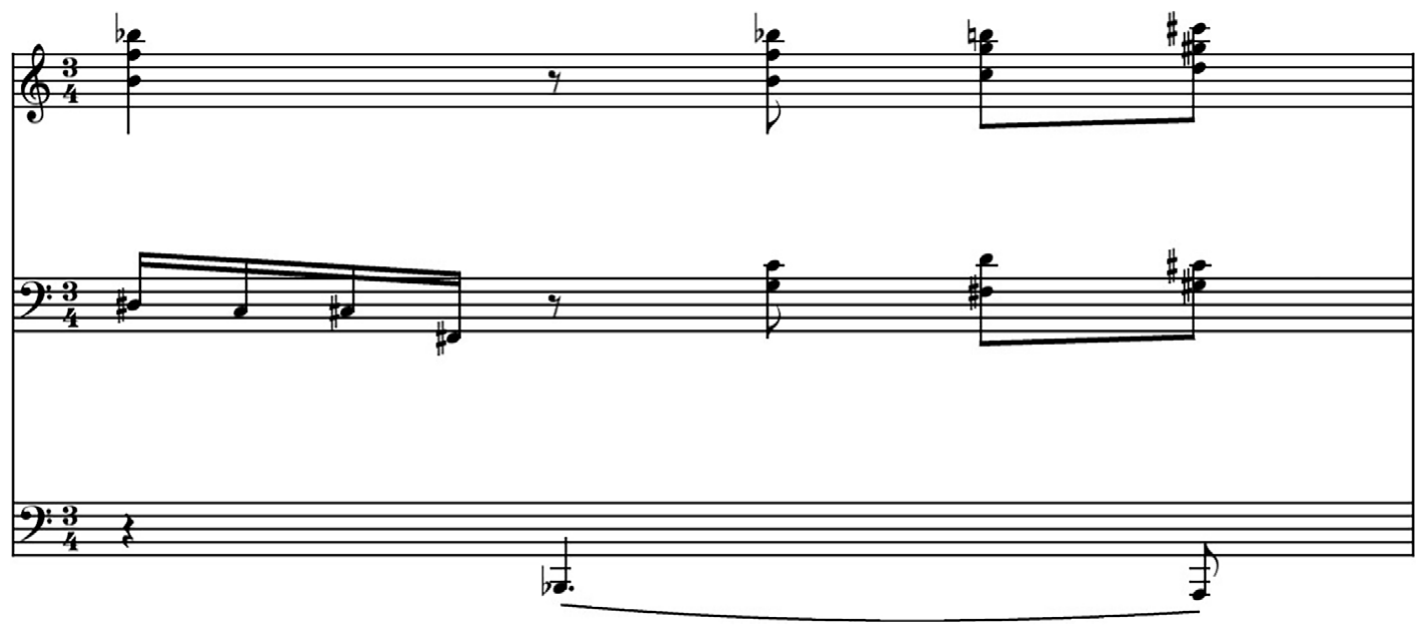
Original music used as a prompt for the AI model.

**Figure 5.**
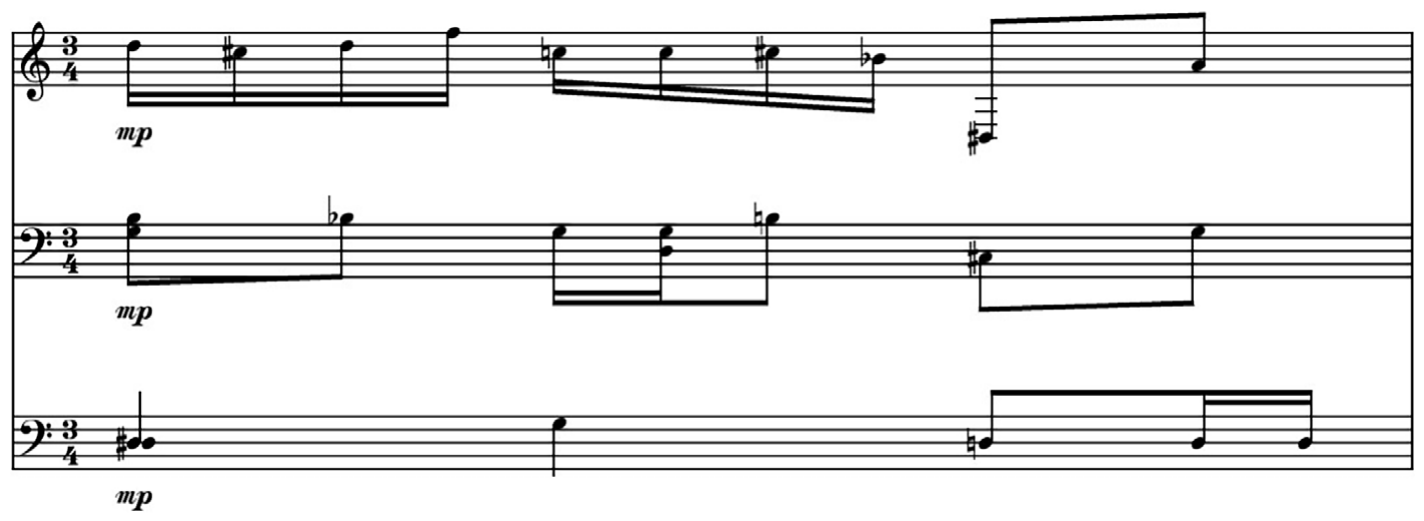
AI generated score based on the prompt from Figure 4.

**Figure 6.**
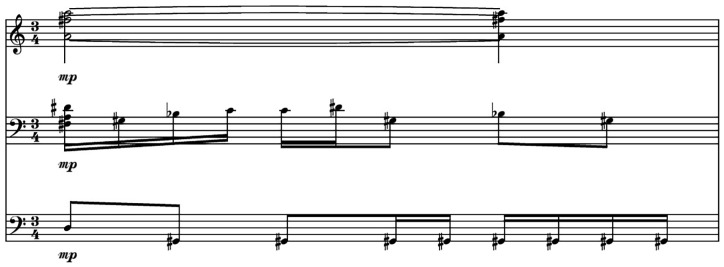
Another AI generated score based on the prompt from Figure 4.

In this section we present score excerpts that have resulted in this second iteration of this research. Figure 4 shows a bar of original music composed by the first author and used as a prompt for the Notochord model. Figure 5 and Figure 6 present content generated by the Notochord model based on the same prompt. As already mentioned in this paper, the durations produced by the Notochord model have been quantized to fit the duration of the generated bar. The quantization unit used in these examples is the 16th note.

We can see that using the same prompt, we get two different outputs. This is useful in case the available content for prompting the model is short. A feature of the Notochord model is that it can feed itself with its own output. With this feature, it is possible to create longer compositions based on a single bar. This would restrict the variety in the context of using the same staves for all generated bars. For example, if the feed contains notes for two of the three staves only, and a rest for the third, all generated output will have a rest for the third staff. For this reason, it is better to have more than just one bar of music to use as a prompt.

The example of Figure 5 took 0.96 seconds to be generated and typed in the LiveLily system, while the example of Figure 6 took 1.7 seconds. The second generated bar incurred a lot of recursion iterations in the quantization process, hence the longer duration for generating it. We can see that the Notochord model is very responsive in a live performance scenario, where, even when combined with the LiveLily system, the typing delay used, and the recursive iterations that might occur, it is still responsive enough to keep a steady pace of an evolving composition. We should of course take into account the tempo of the generated music. In the examples used, the tempo of the original bar is 60 BPM. For a 3/4 meter bar, it takes exactly three seconds for a bar to finish, which is longer than the time Notochord took to generate each of the two bars of Figure 5 and Figure 6, but even with faster tempi, it can perform fast enough to keep a steady pace.

## Conclusions

We have presented the Postdoc research of the first author, titled *Composing for Acoustic Robots - Instant Synthesis for Computer-Controlled Acoustic Instruments Through Live Coding and AI*, with the second author being the scientific supervisor. This report summarizes this research, as it focuses on the second and last iteration, of a series of instant compositions for computer-controlled acoustic instruments. The first iteration has already been presented both in a concert and through a journal article. The instrument used was a Yamaha Disklavier robotic piano. The second iteration used a MIDI-controlled church organ. This paper focused on the development of the technology used for generating music content during the performance, where the AI part of this technology was drastically changed.

Through this research we have developed a system and a method for creating instant compositions for computer-controlled acoustic instruments by creating a traditional Western-music score through live coding. The AI is used to provide music content fast enough so that a steady pace is kept throughout a performance. The content created by the AI resembles its training or feed data, which is original and composed especially for this research.

Besides investigating technology that enables the use of original compositions to be used as a training dataset or feeds to the AI model used, we investigated how to minimize the training procedure, as well as the process of collecting and assembling data to train or feed the AI model. While in the first iteration of this research, we collaborated with four composers who contributed with original music that was used as a training dataset, and we gave credit to their work both in concert in literature, in the second iteration we used the Notochord model, which is trained on the Lakh MIDI dataset. Even though this dataset is comprised of 176,581 MIDI files the composers of which we did not collaborate explicitly, we are able to provide a link to an online list of meta data on these files, that concern composer names, and other credit data.

## Ethics and consent

Ethical approval and consent were not required

## Data Availability

In this section, the data used in this research are provided. LiveLily software Source code available from: https://github.com/alexdrymonitis/LiveLily/tree/develop Archived software available from: 10.5281/zenodo.16020410 ( Drymonitis, 2025a). Licence: MIT Notochord software Source code available from: https://github.com/Intelligent-Instruments-Lab/notochord Archived software available from: 10.5281/zenodo.16116596. ( Shepardson & Armitage, 2025) Licence: MIT COMACROB software Source code available from: https://github.com/alexdrymonitis/comacrob Archived software available from: 10.5281/zenodo.16020691 ( Drymonitis, 2025b). Licence: MIT

## Footnotes

^1^ https://github.com/Skuldur/Classical-Piano-Composer/

^2^ https://iil.is/

^3^ https://colinraffel.com/projects/lmd/
