# Supplementary material for: Composing for acoustic robots: Instant synthesis for computer-controlled acoustic instruments through live coding and AI – A research conclusion
Source: Open Res Eur. 2025 Aug 4;5:216. [Version 1] doi: 10.12688/openreseurope.20968.1 (PMC12754358; doi:10.12688/openreseurope.20968.1)
Supplement: Peer review history

# Reviewer report: Reviewer response for version 1

**DOI:** 10.21956/openreseurope.22685.r64751

**Published:** 2025-12-30

**Authors:** adam patrick bell

**Affiliations:** 1 Western University, London, Canada

In summary, this article presents one aspect of a larger research study about "instant composition" with a system that uses live coding and AI. Perhaps the authors are limited by word count or conventions of this medium, but I anticipated the inclusion of a rationale for the study (i.e., why does this research need to be done? Who does it benefit and how?). All of the references in the opening section are to the first author, which makes it challenging for a reader to know the broader context of this study, such as how it is situated within the field and amongst related studies. These issues aside, overall, the paper presents original work and contributes new knowledge to the field of music technology, and more specifically, the area of new musical interfaces.

If any, are all the source data and materials underlying the results available?

Yes

Is the work original in terms of material and argument?

Yes

Is the work clearly and cogently presented?

Yes

Is the argument persuasive and supported by evidence?

Yes

Does the research article contribute to the cultural, historical, social understanding of the field?

Yes

Does it sufficiently engage with relevant methodologies and secondary literature on the topic?

Partly

Reviewer Expertise:

music education, music technology

I confirm that I have read this submission and believe that I have an appropriate level of expertise to confirm that it is of an acceptable scientific standard.

# Reviewer report: Reviewer response for version 1

**DOI:** 10.21956/openreseurope.22685.r63572

**Published:** 2025-11-20

**Authors:** Stephanie Ness

**Affiliations:** 1 Diplomatic Academy Vienna, Vienna, Vienna, Austria

Your research on creating instant synthesis for computer-controlled acoustic instruments uses AI to generate fully notated music. It is novel in that it is allowing traditional Western scores to evolve dynamically during performance. From my understanding, you have short instant compositions for a Yamaha Disklavier robotic piano. The AI trained on four original atonal compositions then generated harmonic content only, while you manually controlled the rhythm of the final works. In a second iteration, you used an instant composition for a MIDI-controlled church organ using the Notochord AI model, trained on the Lakh MIDI dataset. From my understanding you do something similar as the Shepardson (2024). https://arxiv.org/abs/2403.12000 

 I have one suggestion regarding the paper that can increase its reach by critically engaging with a larger audience. The research article would in my opinion benefit from also answering the following question with reference to 2-3 references: What does it mean aesthetically or philosophically to “compose with robots”? And how does this relate to existing literature in digital performance, in particular AI scoring in music? Maybe you could add a paragraph: Existing literature after the Introduction section.

If any, are all the source data and materials underlying the results available?

Yes

Is the work original in terms of material and argument?

Yes

Is the work clearly and cogently presented?

Yes

Is the argument persuasive and supported by evidence?

Partly

Does the research article contribute to the cultural, historical, social understanding of the field?

Yes

Does it sufficiently engage with relevant methodologies and secondary literature on the topic?

Yes

Reviewer Expertise:

Cybersecurity & Adversarial Robustness, live scoring, artificial intelligence, critical infrastructure, creative industries and education, Ethics, Trust, Privacy and Responsible AI.

I confirm that I have read this submission and believe that I have an appropriate level of expertise to confirm that it is of an acceptable scientific standard.
